# Platelet Rich Plasma—A Healing Aid and Perfect Enhancement Factor: Review and Case Report

**DOI:** 10.5005/jp-journals-10005-1085

**Published:** 2011-04-15

**Authors:** Rani Somani, Iram Zaidi, Shipra Jaidka

**Affiliations:** 1 Professor, Department of Pediatric and Preventive Dentistry, DJ College of Dental Sciences and Research Modinagar, Ghaziabad, Uttar Pradesh, India; 2 Postgraduate Student, Department of Pediatric and Preventive Dentistry, DJ College of Dental Sciences and Research Modinagar, Ghaziabad, Uttar Pradesh, India; 3 Associate Professor, Department of Pediatric and Preventive Dentistry, DJ College of Dental Sciences and Research Modinagar, Ghaziabad, Uttar Pradesh, India

**Keywords:** Growth factor, PRP, Regeneration, Wound healing.

## Abstract

Platelet rich plasma (PRP) has been a breakthrough in the stimulation and acceleration of tissue healing. It represents a relatively new approach in regenerative procedures and is a developing area in pediatric dentistry. It is an autologous source of growth factors obtained by sequestrating and concentrating platelets by gradient density centrifugation. This novel and potentially promising technique enhances body’s natural wound healing mechanism. This article goes on to describe preparation and clinical benefits of PRP in pediatric dentistry.

## INTRODUCTION

Platelet rich plasma, a novel based biologically active tool, is a new approach in pediatric dentistry. Platelet rich plasma brings the power of modern biological, chemical and physical science to solve the real clinical problems in pediatric dentistry. Marx in 1998 introduces PRP for reconstruction of mandibular defects, and it represents a relatively new biotechnology that is part of the growing interest in tissue engineering and cellular therapy. Gibble and Ness in 1990 introduced fibrin glue, alternatively referred to as fibrin sealant or fibrin gel, a biomaterial that was developed in response to the necessity for improved hemostatic agents with adhesive properties. Platelet rich plasma gel (PRP gel) is an autologous modification of fibrin glue obtained from autologous blood used to deliver growth factors in high concentrations. It is an autologous concentration of human platelets in a small volume of plasma, mimics coagulation cascade, leading to formation of fibrin clot, which consolidates and adheres to application site. Its biocompatible and biodegradable properties prevent tissue necrosis, extensive fibrosis and promote healing.

### Availability of PRP

It is an autologous source of growth factors obtained by sequestrating and concentrating platelets by gradient density centrifugation.

### Constituents of PRP

PRP contains high concentration of platelets and native concentration of fibrinogen. The alpha granules of platelets include a high concentration of factors, which are released on activation of platelets by adding calcium chloride and thrombin to PRP. The growth factors are diverse group of polypeptides that have important roles in the regulation of growth and development of a variety of tissues. Various growth factors released from PRP are listed in Table 1.

## PREPARATION OF PRP

**Platelet Rich Plasma Procurement Techniques**

It can be done by using various techniques:

- Gradient density cell separators
- Concentrating cell separators.

1.   *Using gradient density cell separators:* These are general purpose cell separators like ELMD-500 (Medtronic Electromedic, Autotransfusion System, Parker, CO) require large quantity of blood (450 ml) and need to be operated in hospital setting. Blood is drawn into a bag containing citrate-phosphate-dextrose anticoagulant. First, the blood is centrifuged in a general purpose cell separator, at 5,600 rpm to separate the platelet poor plasma (PPP) from the red blood cells (RBC) and the PRP (also termed “buffy coat,” which contains the platelets and the leukocytes). Then centrifuged at 2,400 rpm to obtain PRP from the slurry of RBC and PRP. The procurement of PRP with this technique can be accomplished in 30 minutes, and use of the obtained PRP is recommended within 6 hours after being drawn from the patient. Platelet counts of 500,000 to 1,000,000 in the PRP are usually obtained with this plasmapheresis technique. With this processing technique, the remaining erythrocytes and PPP can be returned to the circulation or discarded.

**Table 1:** **Table 1:** Summary of growth factors released from platelets

*Growth factor*		*Molecular properties*		*Source cells*		*Target*		*Action*	
PDGF		Cationic polypeptide (Mr = 30 kda)		Platelets, macrophages, monocytes, endothelial cells, smooth muscle cells.		Fibroblasts, smooth muscle cells, glial cells, macro-phages/neutrophils.		Stimulates chemotaxis/mitogen-esis in fibroblast/glial/smooth muscle cells; regulates collage-nase secretion/collagen synthesis; stimulates macrophage/neutrophil chemotaxis.	
TGF-b		2-chain polypeptide (Mr = 25 kda); 3 different gene products in humans: TGF-P1, TGF-P2, TGF-P3		Platelets, T-lymphocytes, macrophages/monocytes, neutrophils.		Fibroblasts, marrow stem cells, endothelial cells, epithelial cells, preosteoblasts.		Stimulates/inhibits endothelial, fibroblastic, and osteoblastic mitogenesis; regulates collagen synthesis/collagenase secretion; regulates mitogenic effects of other growth factors; stimulates endothelial chemotaxis and angio-genesis.	
PDEGF		53-amino acid poly-peptide (Mr = 6 kda)		Platelets, macrophages, monocytes.		Fibroblasts, endothelial cells, epithelial cells.		Stimulates endothelial chemo-taxis/angiogenesis; regulates collagenase secretion; stimulates epithelial/mesenchymal mitogen-esis.	
PDAF		Acidic polypeptide (Mr = 45 kda)		Platelets, endothelial cells.		Endothelial cells		Increases angiogenesis and vessel permeability; stimulates mito-genesis for endothelial cells by direct or indirect actions; several cytokines and growth factors up-regulate PDAF, including IGF-1, TGF-alpha and beta, PDGF, bFGF, PDEGF, and IL-1 beta.	
IGF-1		Single-chain polypeptide (Mr = 47 kda) 47% homology with insulin.		Osteoblasts, macro-phages, monocytes, chondrocytes.		Fibroblasts, osteoblasts, chondrocytes.		Stimulates cartilage growth, bone matrix formation, and replication of preosteoblasts and osteoblasts; acts as an autocrine and paracrine factor; in combination with PDGF can enhance the rate and quality of wound healing.	
PF-4		Homotetramer (Mr = 29 kda)		Platelets		Fibroblasts, neutrophils		Chemoattractant for neutrophils and fibroblasts; potent antiheparin agent.	

PDGF - platelet-derived growth factor; TFG-b - transforming growth factor beta; PDEGF - platelet-derived epidermal growth factor; PDAF - platelet-derived angiogenesis factor; IGF-1 - insulin-like growth factor 1; PF-4 - platelet factor 4; bFGF - basic fibroblast growth factor.

Advantage

RBCs can be returned back to patient venous blood.

Disadvantages

- Need large volume of blood
- Need large hospital setup.

2.   *Using concentrating cell separators:* Platelet concentrating cell separators are more widely used since equipment can be accommodated in dental clinic setup.The technology permits procurement of PRP using smaller volumes of blood, increasing the platelet concentration and avoiding need of RBC and PPP reinfusion. Two such separators commercially available are Harvest SmartPrep Platelet Concentrate System (HSPCS) (Harvest Technologies, Plymouth, MA) and the 3i Platelet Concentrate Collection System (3i PCCS) (3i Implant Innovations, Palm Beach Gardens, FL). Both platelet-concentrating cell separators are similar in performance and simplicity, and they represent a significant advantage that they requires less time to produce the PRP (15 minutes versus 20 minutes) and less operator intervention and training.

Advantages

- Do not need large hospital setup
- Need small volume of blood.

Disadvantage

RBCs cannot be returned back to patients venous blood.

### Properties of Platelet Rich Plasma

- Increase tissue vascularity through increased angiogenesis.
- Enhancing collagen synthesis
- Enhancing osteogenesis
- Increasing the rate of epithetlial, and granulation tissue production.
- Antimicrobial effect
- Reaction with other material: PRP does not react or interfere with any other restorative material glass ionomer cements or composite resin used as filling material are not affected by it.
- Biocompatibility: Any material that is identified to be used in humans or animals should be biocompatible without having any toxic or injurious effects on biologic tissues and its functions. PRP offers a biologically active substance with the release of growth factor.
- Tissue regeneration: PRP allows regeneration of tissue with the release of growth factors.

The properties of PRP are based on the production and release of multiple growth and differentiation factors upon platelet activation. These factors are critical in the regulation and stimulation of the wound healing process, and they play an important role in regulating cellular processes such as mitogenesis, chemotaxis, differentiation and metabolism. Growth factors interact one with another, consequently forming a cascade of different signal proteins with multiple pathways, ultimately leading to the activation of gene expression and then protein production. Recent reports have suggested that PRP leads to more rapid epithelialization, more dense and mature bone with better organized trabeculae, and greater bone regeneration.

## MECHANISM OF ACTION OF PLATELET RICH PLASMA

Platelet rich plasma has been found to work via three mechanisms:

1. *Increase in local cell division (producing more cells):* According to Nathan E Carlson 2002 after the injury, platelets begin to stick to exposed collagen proteins and release granules containing adenosine diphosphate, serotonin and thromboxane, all of which contribute to the hemostatic mechanism and the clotting cascade.
2. Inhibition of excess inflammation by decreasing early macrophage proliferation.
3. Degranulation of the agranules in platelets, which contain the synthesized and prepackaged growth factors.

The active secretion of these growth factors is initiated by the clotting process of blood and begins within 10 minutes after clotting. More than 95% of the presynthesized growth factors are secreted within 1 hour (Kevy and Jacobson). PRP has been shown to remain sterile and the concentrated platelets viable for up to 8 hours once developed in the anticoagulated state.

## ROLE OF PLATELET RICH PLASMA IN THE PROCESS OF WOUND HEALING

The process of wound healing can be divided into three different stages:

- Biochemical activation
- Cellular activation
- Cellular response.

1. *Biochemical activation* involves the translation of mechanical injury into biochemical signals that can be understood by the body. The trigger that starts the cascades is the Hagemann factor found in serum. When injury causes disruption of the microcirculation, plasma comes in contact with tissue proteins and the basement membrane. This activates the Hagemann factor and circulating platelets. The activated Hagemann factor activates the clotting cascade and produces fibrin to help in hemostasis and thrombin formation that causes the maximal release of platelet alpha granules.
2. *The cellular activation* stage results in the influx of cells into the wound. The first cellular response involves neutrophils, monocytes, and platelets. Platelets accumulate at the wound site in response to the initial injury, in response to thrombin, platelets release their granules that contain locally acting growth factors. These factors signal the local mesenchymal and epidermal cells to migrate, divide, and increase their collagen and glycosaminoglycan synthesis. This initial release is thought to accentuate the reparative response.
3. *The monocytes transformed* into macrophages are involved in the final cellular response. These cells assist the neutrophils in host defense and produce many of the growth factors, which direct repair until the wound is healed.

Platelet rich plasma accentuates all these processes. The active secretion of these growth factors is initiated by the clotting process of blood and begins within 10 minutes after clotting. More than 95% of the presynthesized growth factors are secreted within 1 hour (Kevy and Jacobson). PRP has been shown to remain sterile and the concentrated platelets viable for up to 8 hours once developed in the anticoagulated state. Thus, activated autologous platelet rich plasma releases growth factors that increase collagen content, accelerate epithelial and epidermal regeneration, promote angiogenesis, enhance wound strength, hasten hemostasis, improve tissue regeneration, hasten remodeling, reduce pain, and reduce infection, which ultimately leads to regeneration.

## THERAPEUTIC POTENTIAL OF PRP IN PEDIATRIC DENTISTRY

Platelet rich plasma has been a breakthrough in the stimulation and acceleration of tissue healing. It represents a relatively new approach in regenerative procedures.

Following are the clinical applications of PRP in pediatric dentistry:

1. *Pulp Capping:* PRP has been proposed as a potential medicament for capping of pulps with reversible pulpitis because of its excellent tissue compatibility. It is much superior to routinely used calcium hydroxide based on tissue reaction between these material.
2. *Pulpotomy:* Formocresol has been routinely used as a pulpotomy agent for deciduous teeth. But this material has been criticized for its tissue irritating, cytotoxic and mutagenic effects. PRP was found to be an ideal material with low toxic effect, increased tissue regenerating properties and good clinical results. Damle et al in 2004 compared PRP and calcium hydroxide as pulpotomy agent and found that PRP gives 100% success rate as compared to 60% of calcium hydroxide.
  Nakashaki et al in 2007 compared PRP and hydroxipatite and found that PRP is a better pulptotomy agent.
3. *Extraction Socket:* Extraction has always been a source of trauma, anxiety and fear in children. Therefore in an attempt to promote rapid healing application of PRP was done in extraction socket. Following tooth removal bone formation normally takes 16 weeks and may result in less than adequate volume for the necessary reconstruction. Platelet rich plasma (PRP) has been promoted as an effective method for improving bone formation. Its use is often expensive, time consuming, or not clinically convenient for the patient and/or clinician. PRP initiates healing by sequestrating platelets and enriching natural clot which initiates a rapid and complete healing process. It also accelerates healing by promoting rapid revascularization and re-epithelization of flaps and cell proliferation. Ratushki et al in 2008 has done a study in which they examine a simple method for obtaining a “Buffy Coat”-PRP (BC-PRP) and its effect on bone healing following the removal of bilateral mandibular 3rd molars and found that after application of PRP there was better bone regeneration and better healing.

## CASE REPORTS

Platelet rich plasma has been a breakthrough in the stimulation and acceleration of tissue healing. It represents a relatively new approach in regenerative procedures. Based on this, application of PRP in pulpotomy and extraction socket was done in the Department of Pediatric Dentistry, DJ Dental College, Modinagar. The patients were selected from the outpatient department with good general health, no history of systemic illness or hospitalization and with no history of antibiotic intake in the past six months. The parents and guardians of the child were informed about the status of the child’s dentition and written consent was obtained from them.

## CASE 1

### Pulpotomy with PRP

Criteria for case selection:

The criteria given by Hellig J et al in 1984 and Waterhouse et al in 2000 were followed:

- Teeth with deep carious lesion (radiographically the caries should be approximating to the pulp)
- Teeth should be restorable after completion of the procedure
- Absence of symptoms indicative of advanced pulpal inflammation, such as spontaneous pain or history of nocturnal pain.
- Absence of clinical sign or symptoms suggesting a non-vital tooth such as suppurating sinus soft tissue swelling.
- Absence of clinical radiographic signs of pulpal necrosis, i.e. furcation involvement, periapical pathology, internal resorption, calcification in canal.
- Hemorrhage should stop within five minutes from amputated pulp stumps using a sterile pledget of moist cotton. After assessment of clinical and radiographic criteria, single visit pulpotomy was performed on selected teeth.

## PREPARATION OF PRP

**Fig. 1:**
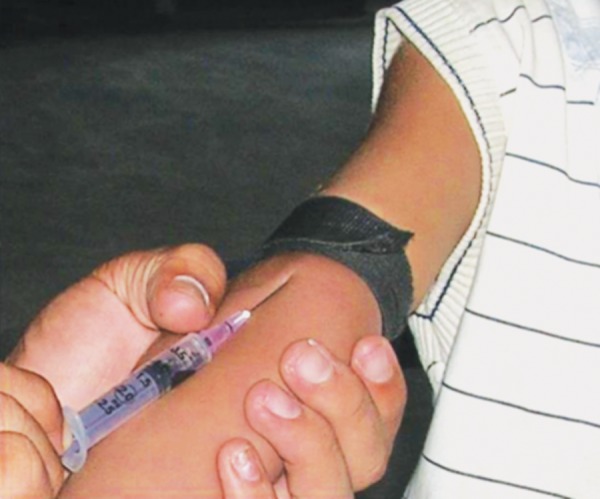
Patient’s venous blood is drawn

**Fig. 2:**
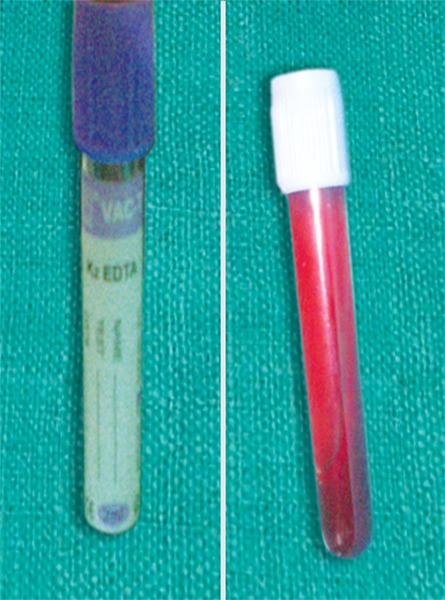
Collected in vaccutube

**Fig. 3:**
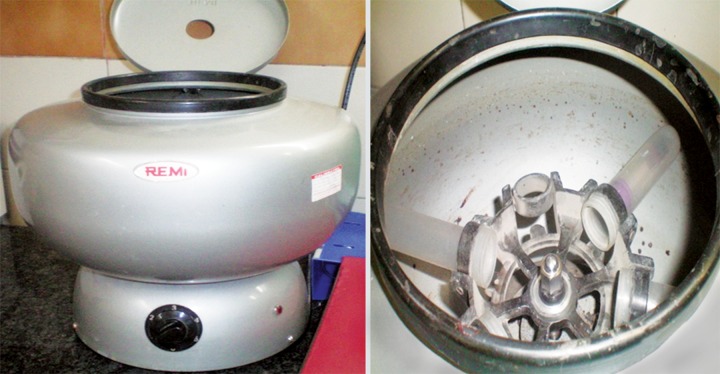
Centrifugal machine

**Fig. 4:**
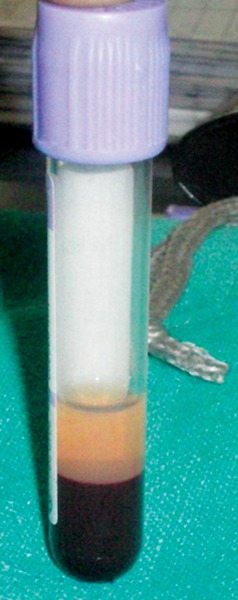
Three layers of PRP

10 ml of patients venous blood is drawn (Fig. 1) and collected in sterile plastic vaccutube coated with anti-coagulant EDTA (Fig. 2). Centrifugation machine (Fig. 3) is used for obtaining PRP with a speed of 1000 rpm for 10 min. After centrifugation three layers are obtained (Fig. 4).

- Upper straw colored fluid PPP (platelet poor plasma)
- Middle buffy coat rich in platelets
- Lower layer rich in RBCs.

This is called soft spin.

**Fig. 5:**
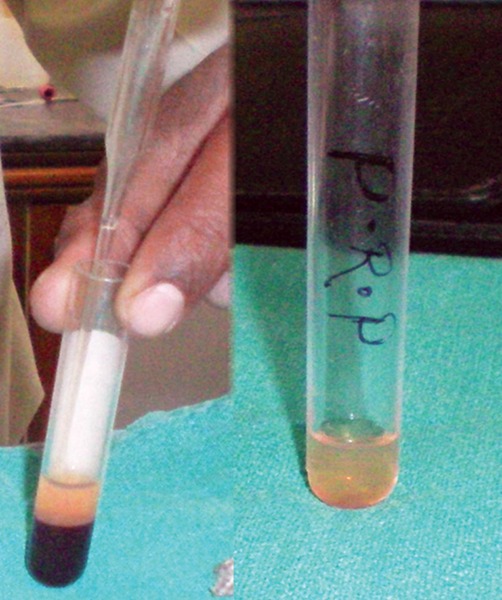
Aspiration of plasma and RBC

**Fig. 6:**
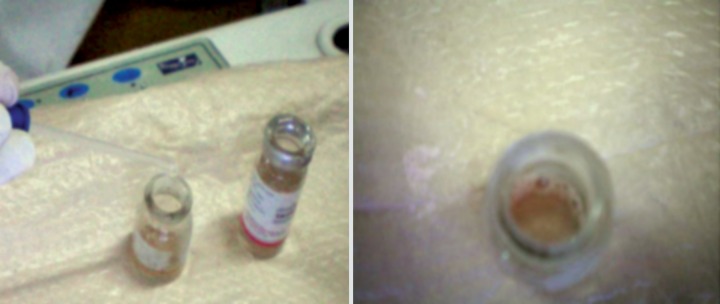
Mixed with calcium chloride

The straw colored plasma and RBC is aspirated and collected in other test tube (Fig. 5), and again centrifugated for 10 min and PRP obtained is placed in sterile test tube. This is called hard spin. For activation PRP is mixed with calcium chloride (Fig. 6). Because it leads to release of growth factors. PRP then obtained is ready for application.

## PROCEDURE

**Fig. 7:**
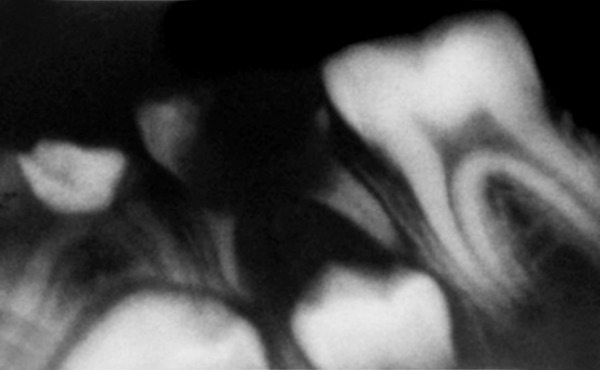
Pulpotomy with PRP

**Fig. 8:**
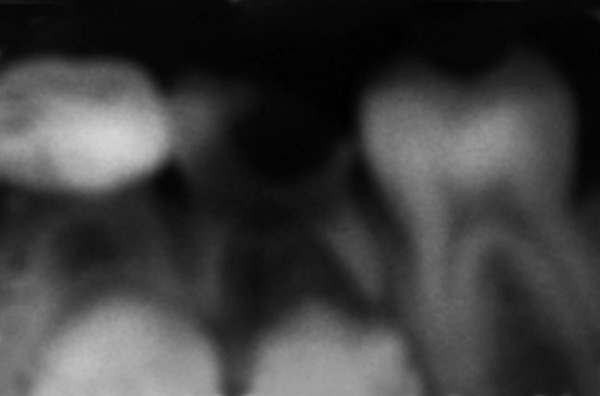
Postoperative after 15 days

**Fig. 9:**
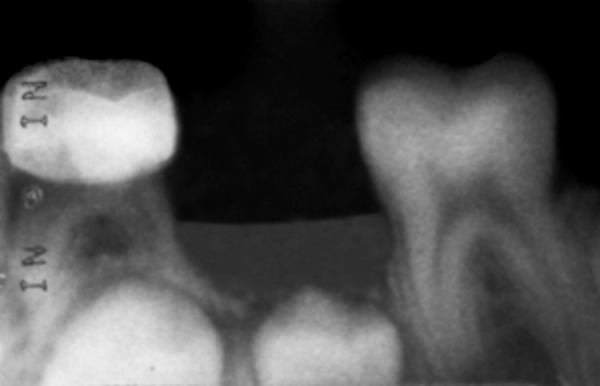
Postoperative after one month

Preoperative radiograph was taken. Local anesthesia was achieved using 2% xylocaine with 1:80,000 adrenaline. The teeth were isolated using rubber dam, cavity outline was established with high-speed round bur with water coolent. Caries was excavated with a spoon excavater. The pulp chamber was entered and roof was removed. After coronal amputation of pulp freshly prepared PRP was placed over the pulp stump and gently packed with the sterile pledget of moist cotton. A thick mix of ZOE was placed to seal the coronal pulp chamber (Fig. 7). Clinical examination was undertaken at 15 days (Fig. 8), 1st and 3rd month intervals, where as radiographic evaluation of the treated teeth were carried out at 1st and 3rd month interval (Fig. 9).

Teeth were evaluated for the presence and absence of following findings:

**Clinical findings:**

- Spontaneous pain or pain initiated by stimuli.
- Signs of sinus formation, tenderness to percussion, soft tissue swelling and mobility.
- Signs of defective restoration or recurrent caries.

**Radiographic findings:**

- Signs of pulpal degeneration, such as periapical or furcal radiolucency, canal calcification, internal resorption.
- Defective restoration or recurrent caries.

## RESULTS

Preoperatively, the incidence of pain was present in all the patients. No signs of pulpal degeneration noticed clinically and radiographically.

Clinical examination after one and three months of treatment revealed absence of pain, swelling and mobility.

Radiographic examination revealed no periapical or furcation involvement. None of teeth showed calcific barrier formation at the mesiodistal width of the root canal. Thus, PRP showed a 100% success rate, as all teeth were asymptomatic in a three months evaluation.

## CASE 2

### PRP in Extraction Socket

**Fig. 10:**
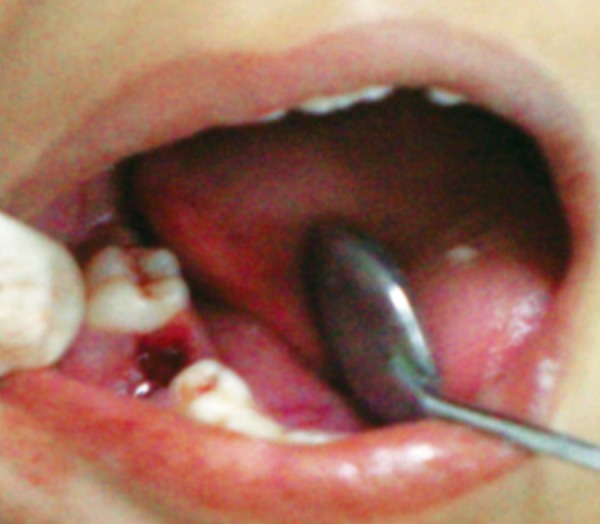
Extraction socket

**Fig. 11:**
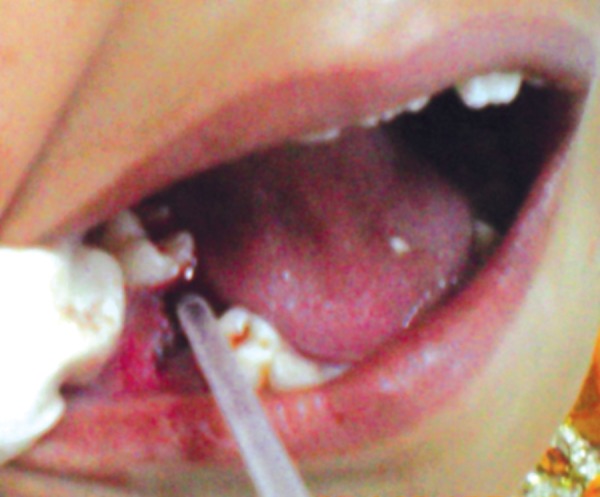
Application of PRP in extraction socket

**Fig. 12:**
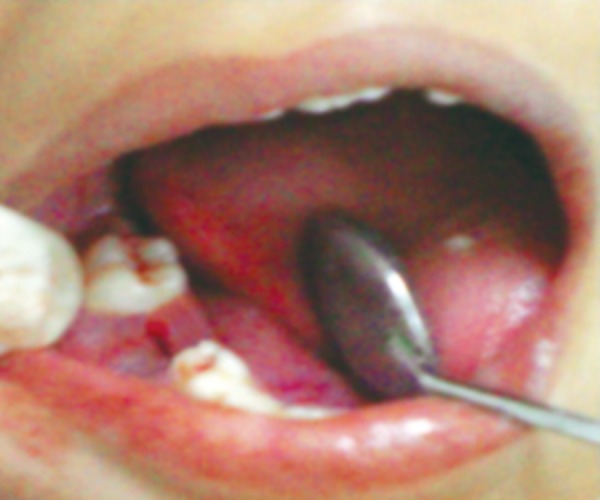
After 3 days (approximation of flaps)

In an attempt to promote rapid healing, freshly prepared PRP was placed in extraction socket immediately after extraction and betadiene pack was given (Figs 10 and 11). Post extraction instructions were given and patient was recalled after 3 days. Clinical examination was done anc it shows rapid healing of socket (Fig. 12). PRP initiates healing by sequestrating platelets and enriching natural clot which initiates a rapid and complete healing process. It also accelerates healing by promoting rapid revascularization and re-epithelization of flaps and cell proliferation.

## CONCLUSION

PRP prepared by sequestration and concentration of platelets is an autologous, safe and user friendly source of growth factors, is an innovative biological tool in the field of pediatric dentistry for pulpotomy and rapid healing of extraction wound.
